# Methane monooxygenases: central enzymes in methanotrophy with promising biotechnological applications

**DOI:** 10.1007/s11274-021-03038-x

**Published:** 2021-03-25

**Authors:** May L. K. Khider, Trygve Brautaset, Marta Irla

**Affiliations:** Department of Biotechnology and Food Science, Norwegian University of Science and Technology, Trondheim, Norway

**Keywords:** Methane monooxygenase, Synthetic methanotrophy, Bioprocesses, Bioremediation, Gas-to-liquid

## Abstract

Worldwide, the use of methane is limited to generating power, electricity, heating, and for production of chemicals. We believe this valuable gas can be employed more widely. Here we review the possibility of using methane as a feedstock for biotechnological processes based on the application of synthetic methanotrophs. Methane monooxygenase (MMO) enables aerobic methanotrophs to utilize methane as a sole carbon and energy source, in contrast to industrial microorganisms that grow on carbon sources, such as sugar cane, which directly compete with the food market. However, naturally occurring methanotrophs have proven to be difficult to manipulate genetically and their current industrial use is limited to generating animal feed biomass. Shifting the focus from genetic engineering of methanotrophs, towards introducing metabolic pathways for methane utilization in familiar industrial microorganisms, may lead to construction of efficient and economically feasible microbial cell factories. The applications of a technology for MMO production are not limited to methane-based industrial synthesis of fuels and value-added products, but are also of interest in bioremediation where mitigating anthropogenic pollution is an increasingly relevant issue. Published research on successful functional expression of MMO does not exist, but several attempts provide promising future perspectives and a few recent patents indicate that there is an ongoing research in this field. Combining the knowledge on genetics and metabolism of methanotrophy with tools for functional heterologous expression of MMO-encoding genes in non-methanotrophic bacterial species, is a key step for construction of synthetic methanotrophs that holds a great biotechnological potential.

## Focus of review

Methane can potentially be used in bioprocesses as it is a low-cost and non-food feedstock and, to some extent, is also considered an unlimited resource. The potential of converting methane to various chemicals and fuels by native methanotrophs has been previously reviewed (Haynes and Gonzalez 2014; Strong et al. 2016; Pieja et al. 2017). We believe it is worthwhile to report the potential of producing MMO in familiar and industrially relevant hosts, especially native and synthetic methylotrophs. For the purpose of this review, synthetic methylotrophs are defined as bacterial strains engineered to be able to utilize methanol as their carbon and energy source, while synthetic methanotrophs are either native or synthetic methylotrophs engineered towards utilization of methane through overproduction of MMO. MMO is known to have a broad substrate profile, including many organic pollutants, and can be applied extensively in the field of bioremediation. Furthermore, it can be used for gas-to-liquid conversion of methane to methanol. The review will cover current knowledge on the potential of methane as a feedstock for bioprocesses, MMOs structural and catalytic properties, attempts at their heterologous production, their applications in biotechnology, and the industrial role of native methanotrophs today. Lastly, we discuss the future of synthetic methanotrophy and its major challenges, and suggest both tested and potential hosts.

## Methane as a feedstock for bioprocesses

Methane, the simplest hydrocarbon and principal component of natural gas, is a powerful greenhouse gas (GHG) with rising atmospheric concentrations (Nisbet et al. 2019). Despite making up only 10% of the greenhouse gases in the atmosphere, methane is 25 times more potent than carbon dioxide (CO_2_) as a GHG, making it a major contributor to global warming (Bera et al. 2009). Methane is naturally emitted from geological sources such as, wetlands, coastal sediments, lakes and oceans, permafrost, and wildfires. A balance exists in the global methane budget, where methane is broken down or consumed in methane sinks. In the atmosphere this is mainly carried out by hydroxyl (OH) radicals through chemical reactions, while methane in the soil on land and sea floor is consumed by methanotrophs. Due to anthropogenic methane emissions from agriculture (paddy cultivation and animal husbandry), fossil fuels and waste (landfill gas) to name a few, a surplus of methane is created that results in an off-balanced global methane budget (Themelis and Ulloa 2007; Lund et al. 2018). While methane in the atmosphere constitutes a concern for global warming and climate change, methane in natural gas plays an important role in fueling engines and turbines, and creating heat. The combustion of methane is more efficient than that of coal and releases half the amount of CO_2_. Thus, labeling it as a ‘bridge’ fuel in the path to achieving lower CO_2_ emissions, but perhaps an even more promising prospective of methane lies in its use as a feedstock in bioprocesses.

In industrial biotechnology, optimization of cell factory design to develop more sustainable production processes remains highly important, however, more focus is placed today on the sustainability of feedstocks and on their conversion to value-added products rather than the production of biomass. In this sense, methane presents itself as a superior feedstock that is both highly abundant and cheap compared to traditional feedstocks (Table 1). In their work, Comer et al. (2017) compared relative costs of methane, glucose, methanol, and acetate as feedstocks in bioprocesses by calculating maximum theoretical yields of different chemical products through flux balance analysis on genome scale metabolic models, and concluded that methane is the most attractive feedstock for microbial cell factories. Methane, unlike sugar cane and molasses, does not compete for resources, such as land and water, with the food industry, making it a sustainable next-generation carbon feedstock (Heux et al. 2018). Additionally, a possible waste-to-product approach may be achieved by utilizing methane generated in landfills, and agricultural or fracking waste. This will depend on advances within the technology of methane capture.

**Table 1:** Global market prices for commodities used as feedstock in industrial biotechnology

Carbon source	Price (USD per metric tonne)
Methane^a^	133*
Carbohydrates	
Glucose^b^	220–661 $/T
Molasses^c^	150–250 $/T
Saccharose^d^	446 $/T
Fructose^c^	1000 $/T
Methanol^e^	492 $/T
Formic acid^f^	323 $/T
Sodium acetate / acetic acid^b^	500 $/T

*1 million British thermal units  = 2.03e^−^^8^ million tonnes liquefied natural gas

^a^Henry Hub (January 2021)

^b^Comer et al. (2017)

^c^Ulber and Sell (2007)

^d^ISO (January 2021)

^e^Methanex (January 2021)

^f^CEIC (April 2020)

### Methanotrophs utilize methane as carbon source

**Fig. 1:**
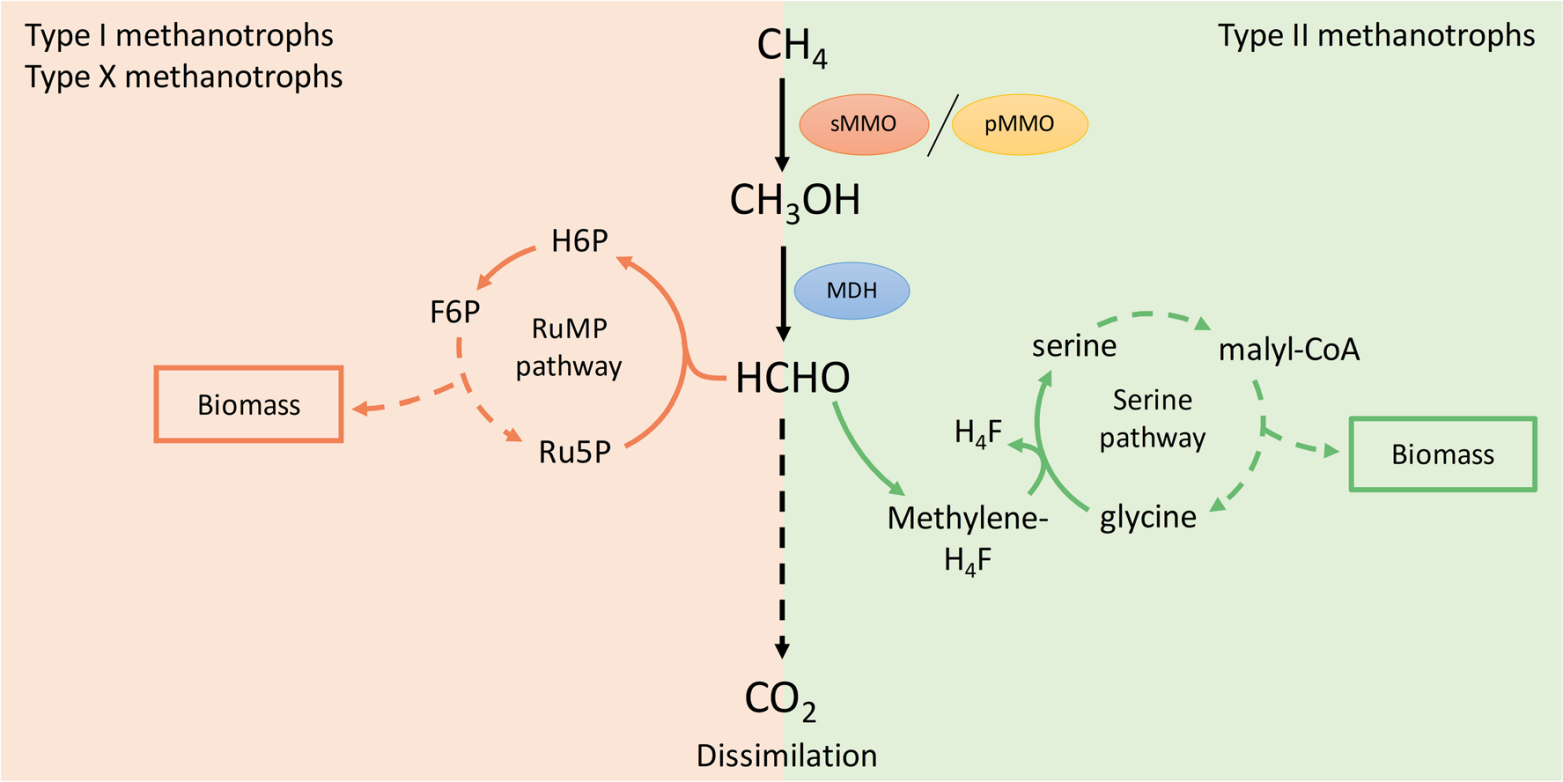
Metabolic pathways of methanotrophs type I, II and X. Methane (CH_4_) is oxidized to methanol (CH_3_OH) by action of soluble methane monooxygenase (sMMO) or particulate methane monooxygenase (pMMO). Methanol dehydrogenase (MDH) then converts methanol to formaldehyde (HCHO). Type I and X methanotrophs utilize the RuMP pathway (orange) to assimilate formaldehyde to biomass. Type II methanotrophs utilize the serine pathway (green) for formaldehyde assimilation. All types of methanotrophs also have formaldehyde dissimilation pathways (middle). Full arrows indicate a single process and dashed arrows indicate several reactions. Abbreviations: RuMP, ribulose monophosphate; H6P, hexulose-6-phosphate; F6P, fructose-6-phosphate; Ru5P, ribulose-5-phosphate; H4F, tetrahydrofolate; malyl-CoA, malyl co-enzyme A

Aerobic methanotrophs are Gram-negative bacteria that are able to utilize methane as their sole source of carbon and energy. First described more than a century ago, they have been the subject of studies since the 1970s (Whittenbury et al. 1970). Whittenbury et al. (1970) described the existence of two different intracytoplasmic membrane structures among different species, leading to the separation of methanotrophs into two types, *Gammaproteobacteria* (type I) and *Alphaproteobacteria* (type II). It later became apparent that the two types exhibit differences in their metabolic pathways for methane assimilation. Type I methanotrophs, containing stacked intracytoplasmic membranes (ICM), assimilate methane oxidised to formaldehyde through the condensation of formaldehyde with a sugar molecule via the ribulose monophosphate (RuMP) cycle (Trotsenko and Murrell 2008). While type II methanotrophs, contain peripherally distributed ICM and assimilate formaldehyde via the serine cycle (Fig. 1). A third type, belonging to the *Gammaproteobacteria* class are type X methanotrophs that assimilate carbon through the RuMP pathway, but also produce enzymes of the serine pathway (reviewed in Hanson and Hanson 1996). Although the *Proteobacteria* are considered elemental participants in many ecosystems, several other methane oxidizers exist that are facultative methanotrophs, extremophiles and anaerobic methanotrophs (reviewed in Kalyuzhnaya et al. 2019).

## Methane monooxygenases are key enzymes for methanotrophic lifestyle

Regardless of their metabolic differences, all aerobic methanotrophic bacteria oxidize methane to methanol with oxygen using the enzyme MMO. To enable methane oxidation in methanotrophs, MMO, through several steps and intermediates, must catalyse the cleavage of two important chemical bonds; the dioxygen bond and the C-H bond of methane (Tinberg and Lippard 2011). There are two types of MMOs; soluble methane monooxygenase (sMMO) and particulate methane monooxygenase (pMMO), whereof methanotrophs can express either or both. The two enzymes are genetically unrelated and while nearly all methanotrophs produce pMMO, only a subset of methanotrophs produce sMMO (reviewed in Dedysh and Knief 2018). In species that produce both enzymes, MMO expression is controlled by a ‘copper switch’ based on copper availability (Stanley et al. 1983). pMMO, a membrane-bound enzyme, uses its copper active site for the oxidation of methane, and is expressed when copper is available, while at a low copper-to-biomass ratio (up to 5.64 µmole Cu^2+^ per gram protein in *Methylosinus trichosporium* OB3b), the cytoplasmic enzyme, sMMO, is active (Morton et al. 2000). In contrast to sMMO, several uncertainties surround the structure and activity of pMMO, such as routes to active site, identity of active sites, O_2_ activation, and electron donors (reviewed in Ross and Rosenzweig 2017). The two MMO forms display different properties, including specific activity, substrate specificity, enzyme stability, and susceptibility to inhibitors (reviewed in Murrell et al. 2000).

sMMO and pMMO will be briefly described hereafter and the reader is referred to more recent and comprehensive papers on their structure and biochemistry (Banerjee et al. 2019; Ross et al. 2019; Ro et al. 2019; Kim et al. 2019; Lieven et al. 2018).

### The mode of action of particulate methane monooxygenase is still not fully elucidated

**Fig. 2:**
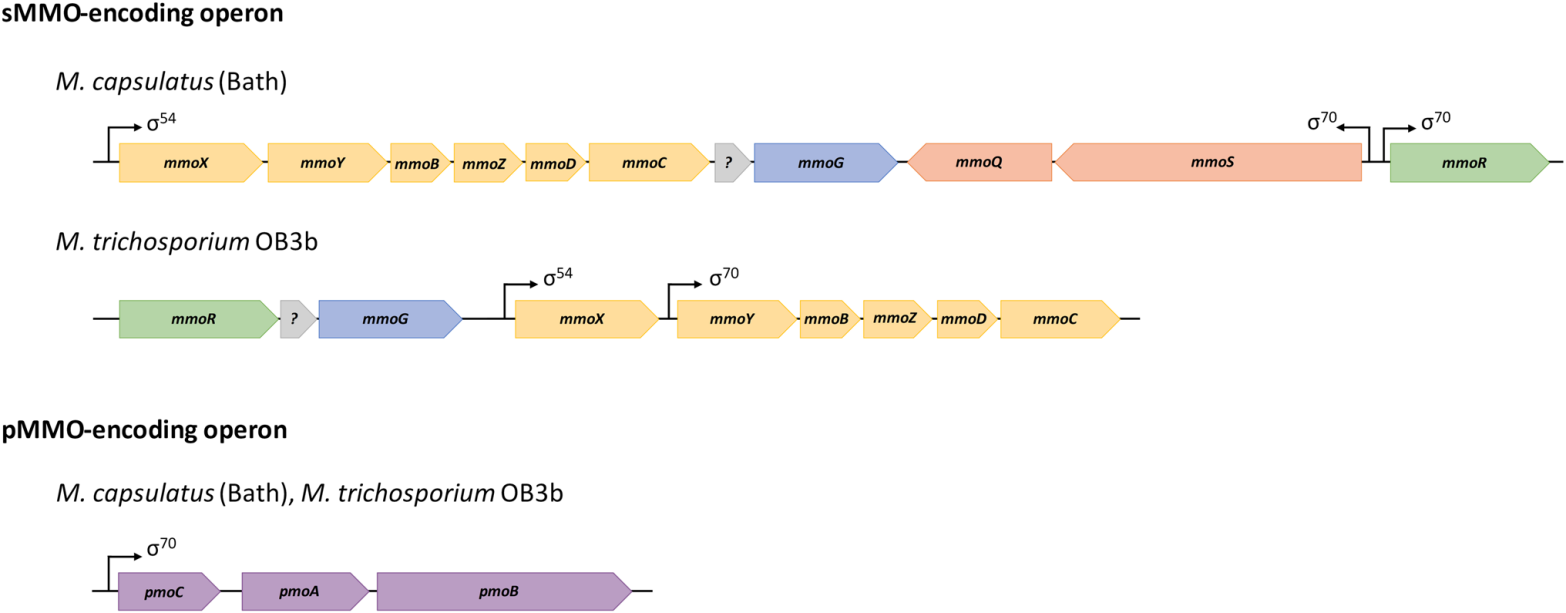
sMMO- and pMMO-encoding operons of *M. capsulatus* (Bath) and of *M. trichosporium* OB3b. Six genes (yellow) encode sMMO. *mmoX, mmoY* and *mmoZ* encode respectively the α-, β- and γ-subunit of the hydroxylase MMOH. *mmoB* encodes the regulator protein MMOB. *mmoD* encodes a disputed regulatory protein MMOD. *mmoC* encodes the reductase MMOC. *mmoG* (blue) encodes MMOG, a GroEL-like chaperonin; *mmoQ* and *mmoS* (orange) encode a two-component sensor system believed to be responsible for the copper-switch; and *mmoR* (green) encodes a reductase MMOR. Hypothetical protein / unidentified open reading frame (grey). Three genes *pmoC, pmoA* and *pmoB* (purple) encode the three subunits of pMMO, PmoA, PmoB and PmoC, respectively. Arrows show promoter sites and indicate the family of sigma factors associated with them (Cardy et al. 1991; Holmes et al. 1995; Ward et al. 2004; Lieberman and Rosenzweig 2004)

Although being the primary metabolic enzyme of methanotrophs, it has been difficult to elucidate the structure of pMMO and characterize it, resulting in several conflicting results over the years (Lieberman et al. 2003; Basu et al. 2003; Balasubramanian et al. 2010; Chen et al. 2014; Ross et al. 2019). This is mainly due to problems in solubilizing and purifying the membrane-bound enzyme. In 2005, the first crystal structure of pMMO of *Methylococcus capsulatus* (Bath) was determined, and the enzyme is now believed to form an *α*_3_*β*_3_*γ*_3_ trimer structure of 300 kDa (Lieberman and Rosenzweig 2005). The trimer subunits PmoA and PmoC are transmembrane domains, while PmoB is a periplasmic domain of the enzyme. In 2019, Ross et al. reported that in pMMO only mononuclear copper active sites are responsible for methane oxidation, of which there are mainly two; Cu_B_ of the soluble subunit PmoB and Cu_C_ of the membrane-bound subunit PmoC. Arranged in the order *pmoCAB,* two nearly identical sets of these pMMO-encoding genes have been found in the genome (*M. capsulatus* Bath), as well as a third copy of *pmoC* (Fig. 2) (Stolyar et al. 1999).

Due to difficulties understanding the role of the intracytoplasmic membrane in the activity of pMMO, its recombinant expression has so far been unsuccessful. In 2000, it was reported by Murrell et al. that expressing parts of the *pmo* gene cluster had toxic effects on *Escherichia coli*. In addition, there is the common issue of incorrect subunit assembly. In their study, Balasubramanian et al. (2010) reported that a recombinant soluble part of PmoB (*spmoB*) binds copper and displays typical MMO activity by demonstrating propylene and methane oxidation and proposed that Cu_B_ was the active site of pMMO. However, the activity of this truncated subunit was later revealed to be the activity of duroquinol (artificial electron donor), which through a series of chemical reactions following Fenton and Haber–Weiss chemistry, was able to oxidize methane (Ross et al. 2019). In active form, pMMO has been reported to display 10 to 100-fold loss in activity after lysis, solubilization and purification from membranes (Sirajuddin and Rosenzweig 2015; Lawton and Rosenzweig 2016a). An activity of ≲ 17% compared to in vivo environment has been reported after isolating pMMO from its native membranes, with the loss in activity suggested to be the result of change of the essential metallocofactor (Ross et al. 2019).

### Soluble methane monooxygenase is a complex multimeric enzyme

Over 20 years of studies have focused on characterizing sMMO from mainly two sources; *Methylococcus capsulatus* (Bath) (type X) and *Methylosinus trichosporium* OB3b (type II) (Foster and Davis 1966; Whittenbury et al. 1970). Although representing different bacterial genera, they possess very similar sMMOs. sMMO belongs to a large family of bacterial multicomponent monooxygenases (BMMs) and is the only BMM to oxidize methane. The enzyme requires three protein components for its catalytic activity; a hydroxylase (MMOH), a reductase (MMOR) and a regulatory protein (MMOB). The hydroxylase forms a soluble 245 kDa *α*_2_*β*_2_*γ*_2_ homodimer composed mostly of *α*-helices (Fox et al. 1989). Each monomer in MMOH contains a well-characterized, carboxylate-bridged, nonheme binuclear iron active site, located in the α subunit (Fox et al. 1989; Tinberg and Lippard 2011). The diiron active site catalyses the activation of O_2_ and hydroxylation of methane (reviewed in Wang et al. 2017). The second component of the enzyme, MMOR, is a 40 kDa nicotinamide adenine dinucleotide (NADH)-dependent and flavin adenine dinucleotide (FAD)-containing reductase that delivers two electrons to the active site (Banerjee et al. 2019). Lastly, when the regulatory component, MMOB, a 16 kDa protein binds to MMOH stoichiometrically the reaction rate of MMOH increases by 1000 fold (Liu et al. 1995).

sMMO-encoding genes are found in a six-gene operon with arrangement *mmoXYBZDC*. *mmoX, mmoY* and *mmoZ* encoding the *α*-, *β*- and *γ*-subunits of the hydroxylase, respectively (Fig. 2). Within the six-gene operon *mmoB* and *mmoC* encode MMOB and MMOR, respectively (Fig. 2). *mmoD*, formerly *orfY*, an open reading frame of previously unknown function, encodes a protein of disputed function, which is now believed to be a regulatory protein that interacts directly with MMOH (Semrau et al. 2013; Kim et al. 2019). Several genes adjacent to the sMMO operon have been described, such as *mmoG* and *mmoR*, encoding a GroEL-like chaperone and a transcriptional regulator, respectively (Csáki et al. 2003). The mutagenesis of these genes was shown to inhibit *mmo* transcription and resulted in non-functional sMMO (Stafford et al. 2003). Downstream of the sMMO operon in *M. capsulatus* (Bath), a two-component signalling system consistent of a regulator encoded by *mmoQ* and sensor encoded by *mmoS* has been proposed to play a role in the copper switch (Fig. 2) (Csáki et al. 2003; Ukaegbu et al. 2006; Ukaegbu and Rosenzweig 2009).

The catalytic cycle of sMMO starts with the reduction of iron ions from Fe(III) to Fe(II) by MMOR. Next, the diiron center reacts with O_2_ to form a diiron(III)-peroxo intermediate. The key step that follows, cleaving the O–O bond, results in the formation of the compound Q, which maintains both iron atoms in an oxidation state of + 4. Intermediate Q plays the key role in the conversion of methane to methanol by breaking the C–H bond in methane (Lee et al. 1993; Tinberg and Lippard 2011). The structure of Q has been disputed, with initial studies suggesting that the two iron ions are bridged by oxygen atoms to form a “diamond core”, and succeeding research indicating an open-core structure (Shu et al. 1997; Banerjee et al. 2015; Castillo et al. 2017; Cutsail et al. 2018). In vivo, sMMO has high levels of activity with conversion rate of methane estimated to be at least 800 nmoles min^−1^ mg^−1^ (Fox et al. 1989). Fox et al. (1989) reported that the purified sMMO components MMOH, MMOR and MMOB alone have specific activity rates of 1700, 26,100 and 11,200 nmol min^−1^ mg^−1^, respectively.

## Applications of native methanotrophs and methane monooxygenases in biotechnology

Methanotrophs have a primary role in soil methane sinks. Methanotrophs could, due to the extraordinary catalytic capabilities of MMOs, play other roles; a vehicle for methane utilization in gas-to-liquid (GTL) processes, bioremediation and the production of value-added compounds. The interest in exploiting methanotrophs for bioprocesses has increased research activities in recent years, with aims to produce fuels, single-cell proteins, biopolymers, supplements, vitamins, methanol, formaldehyde, propylene oxide, organic acids, enzymes and other chemicals (reviewed in Strong et al. 2015).

Use of methanotrophs in the industry today is limited to the generation of biomass for animal feed from single cell protein (Biswas et al. 2020; Tsapekos et al. 2020; Øverland et al. 2010). Companies like Calysta and Unibio have successfully industrialized and commercialized gaseous fermentation by methanotrophs for the generation of feed biomass using a U-loop fermentor (Larsen 2002).

### Broad substrate range of methane monooxygenase allows for its use in bioremediation

sMMO is claimed to be one of the most powerful, biological oxidants. This is mainly due to its wide catalytic specificity, which extends to oxygenation of carbon monoxide, several alkanes, alkenes, ethers, halogenated methanes, and cyclic and aromatic compounds (reviewed in Murrell and Smith 2010). Being a versatile biocatalyst of over 100 documented substrates opens the door for bioremediation applications of MMO for the degradation of pollutants such as trichloroethylene (TCE) and chlorinated hydrocarbons (reviewed in Smith and Nichol 2018). In several studies the broad oxidation spectrum of sMMO for toxic and nondegradable compound has been employed, most notably for TCE degradation. In situ bioremediation based on the degradation of compounds, such as TCE, in the form of biological treatments with mixed methanotrophic communities enriched from the environment has been thoroughly investigated in several studies (Taylor 1993; Sutfin and Ramey 1997; Walter et al. 1997). Pure methanotrophic cultures can be used for ex situ bioremediation, where in a single-stage bioreactor, the MMO-producing biocatalysts are grown and used for biodegradation within the one reactor. Since the degradation process requires the action of the very same enzyme the organism depends on for growth, there is an inevitable competition that can hamper the bioreaction and is solved by multi-stage systems, where the growth and degradation processes occur separately (reviewed in Jiang et al. 2010). Although sMMO is known to have a broader substrate range and higher catalytic efficiency than pMMO, studies have indicated that pMMO may be more adaptable for the implementation of bioremediation, due to sensitivity of sMMO to copper in the environment (reviewed in Park and Kim 2019).

### In vivo use of methane monooxygenases for gas-to-liquid conversion

As stated in the first section, while methane is an abundant feedstock with relatively low price, the cost of its use in industry increases due to the fact that it is dangerous and expensive to handle and transport (reviewed in Rosenzweig 2015). Although it requires costly procedures such as synthesis gas production followed by Fischer–Tropsch (FT) and separation processes, gas-to-liquid (GTL) conversion of methane to methanol is a proposed solution to several problems connected to use of natural gas as methane source (reviewed in Lee et al. 2016). The expensive and inefficient chemical conversion of methane to methanol has prompted many to suggest the use of MMOs in this process, which naturally oxidize methane at ambient temperature and atmospheric pressure (Fei et al. 2014; Haynes and Gonzalez 2014; Henard et al. 2016). This is where methanotrophs, which naturally produce MMOs, come in as a biological catalyst alternative for GTL conversion (Bio-GTL). By inhibiting the activity of methanol dehydrogenase (MDH), which catalyses oxidation of methanol to formaldehyde, accumulation of methanol is possible, but would have negative implications on the growth of facultative methanotrophs (reviewed in Hwang et al. 2018; Park and Kim 2019). This issue can be addressed with use of a multi-stage reactor where growth of biomass is prioritized in the first stage and activity of MDH is inhibited to allow methanol accumulation in the second stage. Bio-GTL has not yet been achieved and the details of such a process still need to be determined.

Despite the lower carbon and energy efficiencies of MMOs compared with methyl-coenzyme M reductase (MCR), and the need for improving the overall growth rates of aerobic methanotrophs and bioreactor design, aerobic pathways are favoured over anaerobic oxidation of methane due to the lack of genetic tools for anaerobic methanotrophs (ANME) and the slow action of ANME MCR (Sirajuddin and Rosenzweig 2015; Lawton and Rosenzweig 2016b). An interesting and more promising alternative to *mdh*-deficient native methanotrophs would be heterologous production of MMO in non-methanotrophic bacteria. This could allow higher growth rates of host organisms and would prevent methanol oxidation. The insight into efforts with regards to heterologous production of MMO are described in section Challenges with establishing functional heterologous expression of methane monooxygenase.

### Challenges to biotechnological applications of native methanotrophs

Advances in genetic tools for methanotrophs have been made and are comprehensively reviewed by Kalyuzhnaya et al. (2015), and Henard and Guarnieri (2018), including mutagenesis and expression studies, the introduction of broad-host range plasmids and use of inducible promoters, whole-genome metabolic models enabling modelling of flux-balance, and novel strains with rapid growth and better cultivation capacity.

While some methanotrophs are able to grow on methanol and multi-carbon sources, they are largely obligate, and this has contributed to the slow progress in developing tools for their engineering (reviewed in Hwang et al. 2014; Kalyuzhnaya et al. 2019). Other obstacles in the utilization of methanotrophs have been slow growth, with growth rates of 0.3–0.4 h^−1^, compared to *E. coli* (0.4–0.7 h^−1^ in minimal media), and reduced ability to grow in pure cultures compared to mixed cultures with heterotrophs (reviewed in Pieja et al. 2017).

Achieving production of chemicals by applying natural methanotrophs has as of yet not been realized despite the extensive research activities. Instead of focusing on engineering methanotrophs however, many scientists have aimed their efforts at establishing heterologous production of MMO to achieve synthetic methanotrophy.

## Future perspectives in applications of heterologously produced methane monooxygenases

Efficiency of gas-to-liquid conversion may be improved if methanol is not further converted, which means that use of *mdh*-deficient organisms is required. The same principle is valid for a bioremediation approach, where only MMO activity is needed, and therefore use of fast-growing, non-methylotrophic, well-established hosts engineered for protein overproduction can be advantageous, examples of such microorganisms are *E. coli* or *Bacillus* species.

### Challenges with establishing functional heterologous expression of methane monooxygenase

As previously mentioned, pMMO is difficult to express heterologously due to its reported toxicity and membrane-bound nature. Therefore, efforts on heterologous MMO expression have mainly focused on sMMO. Heterologous expression studies of sMMO-encoding genes have been a challenge for almost 30 years, yet every attempt has contributed with valuable knowledge on the expression of this complex enzyme. In 1992, West et al. set out to express sMMO-encoding genes from *M. capsulatus* (Bath) in *E. coli* by the use of T7 polymerase expression system, where they reported production of an active MMOB and MMOR, but an inactive MMOH. The suspected reason for the inactive MMOH was a lack of assembly factors in *E. coli* (West et al. 1992). A few years later, Jahng and Wood (1994) and Jahng et al. (1996) documented their attempts at expressing sMMO-encoding genes from *M. trichosporium* OB3b in *Pseudomonas putida* F1, *Agrobacterium tumefaciens* and *Rhizobium meliloti*, but when these recombinant strains were later assayed for sMMO activity, it was insignificant compared to the control (Jahng et al. 1996; Lloyd 1997). Unsuccessful production of sMMO in *E. coli* has been mainly attributed to the incorrect assembly of MMOH. In one study, Lloyd et al. (1999a) reported the homologous expression of an active sMMO from *M. trichosporium* OB3b in *smmo* deficient mutants. The same year, Lloyd et al. (1999b) used the methanotrophs, *Methylomicrobium album* BG8 and *Methylocystis parvus* OBBP, as hosts for heterologous production of sMMO (from *M. trichosporium* OB3b), and successful transcription of the recombinant *smmo* genes under low copper-to-biomass ratio was reported. *M. parvus* OBBP and *M. album* BG8 contain only pMMO and their transformation with a broad-host range plasmid via conjugation enabled expression of the sMMO genes. For 20 years, there has not been a new study on heterologous MMO expression, with the exception of a few patents, filed in 2016 and 2018 (Clarke et al. 2019, 2020), which describe renewed efforts with optimistic results. The patents report the use of several plasmid constructs with different arrangements of *smmo* genes from *M. capsualtus* (Bath) and the results of their expression in *E. coli*, and the testing of MMO activity in the engineered microorganisms. An intriguing report made by Clarke et al. (2019) is the more than tenfold increase in conversion rate by MMO when chaperone proteins were overproduced not only from the source organism (*M. capsualtus* Bath), but also from the host organism (*E. coli*). Additionally, it was reported that the sMMO from *M. capsualtus* (Bath) displayed significantly higher conversion rates for methane and ethane when compared to sMMOs from *Methylocaldum* sp 175 and *Solimonas aquatica* DSM 25927 (Clarke et al. 2019).

As mentioned, MMO is encoded by a complex operon with several genes where several have auxiliary function and may have been overlooked in previous attempts (Fig. 2). The presence of several subunits could be a contributing factor to challenging expression as it would be more sensitive to incorrect protein maturation. It has been pointed out that protein GroEL-like chaperone protein encoded by *mmoG* is essential for the maturation and functional expression of sMMO (Lloyd 1997). Similarly, a GroEL-like chaperonin protein was required for the expression of a binuclear iron monooxygenase of mycobacteria strains (Furuya et al. 2012, 2013).

Another important limitation to be considered, is the inhibiting effect of copper not only on the expression of sMMO-encoding genes, but also on the enzymatic activity of sMMO in cell free fractions (Semrau et al. 2010). This copper sensitivity can be a potential obstacle, although minor, in establishing heterologous production of sMMO. The presence of copper in growth media is common and growth experiments on low copper levels are crucial to determine the viability of using a particular host.

### Synthetic methanotrophy: use of natural and synthetic methylotrophs

Methylotrophs are naturally able to metabolize one-carbon (C1) compounds, such as methanol, for their growth and energy production. Methanol is metabolized to formaldehyde by the action of methanol dehydrogenase and is a very prevalent enzyme in C1 metabolism. In methanotrophs, only one more enzymatic step is present which allows the conversion of methane to methanol. This places methylotrophs as favorable candidates to become synthetic methanotrophs through the introduction of heterologous production of MMO. Methylotrophs are naturally tolerant to the otherwise toxic effects of formaldehyde due to the presence of different linear and cyclic formaldehyde dissimilation pathways. Additionally, the activity of MDH requires an efficient regeneration system for electron acceptors, for example pyrroloquinoline quinone (PQQ) and nicotinamide adenine dinucleotide (NAD), which are present in native methylotrophs, and would have to be engineered into bacteria that do not naturally use methanol as carbon source. The presence of these features facilitates the process of introduction of synthetic methanotrophy to native methylotrophs, making concerns such as accumulation of toxic levels of formaldehyde or the regeneration of MDH co-factors negligible (Wang et al. 2019). The addition of a single new pathway, through introduction of an enzymatic step catalyzed by MMO, is not easy, as previously described. The natural methylotrophs *Baciullus methanolicus* and *Methylobacterium extorquens* are considered feasible candidates for use in industrial processes. For that reason it can be imagined that extending their metabolism with a reaction catalysed by MMO would allow for the creation of methane-based bioprocesses (Ochsner et al. 2015; Brautaset et al. 2007). However, both of those strains currently have specific constraints for widespread industrial use, with lack of tools for genomic modifications for *B. methanolicus* and relatively low growth rates in comparison to commonly used industrial strains for *M. extorquens* (Gil López et al. 2019; Delaney et al. 2013). Therefore, it may be feasible to achieve methane oxidation in synthetic methylotrophs. Recently a lot of progress has been made regarding engineering of different industrially relevant microbial strains, such as *E. coli*, *Bacillus subtilis* and *Corynebacterium glutamicum,* for synthetic methylotrophy (reviewed in Heux et al. 2018; Antoniewicz 2019; Wang et al. 2020). It is beneficial to use established industrial strains because they have been engineered and optimized for production of a wide range of chemicals, the production bioprocesses are well established and they are classified as Generally Recognized As Safe (GRAS), which permits for their use for production of food and feed additives.

A major challenge for establishing synthetic methanotrophy is the possibility of inadequate growth rates and biomass production by synthetic methanotrophs. In the case of both native and synthetic methylotrophs engineered for methane utilisation, the C1 assimilation pathway will support both biomass formation and production of value-added compounds. One of the conditions for methane assimilation to be sufficiently efficient is production of MMO at high enough level. Low MMO activity would lead to low growth rates and low biomass formation. Taken together, despite possible challenges, establishment of synthetic methanotrophy through heterologous MMO production seems to be a feasible alternative to current systems for methane utilisation.
